# Knockdown of CYP24A1 Aggravates 1α,25(OH)_2_D_3_-Inhibited Migration and Invasion of Mouse Ovarian Epithelial Cells by Suppressing EMT

**DOI:** 10.3389/fonc.2020.01258

**Published:** 2020-07-29

**Authors:** Ping Wang, Jiming Xu, Weijing You, Yongfeng Hou, Shuiliang Wang, Yujie Ma, Jianming Tan, Zengli Zhang, Wentao Hu, Bingyan Li

**Affiliations:** ^1^Department of Nutrition and Food Hygiene, School of Public Health, Soochow University, Suzhou, China; ^2^Fujian Key Laboratory of Transplant Biology, 900 Hospital of the Joint Logistics Team, Fuzhou, China; ^3^State Key Laboratory of Cardiovascular Disease, National Center for Cardiovascular Diseases, Fuwai Hospital, Chinese Academy of Medical Sciences and Peking Union Medical College, Beijing, China; ^4^State Key Laboratory of Radiation Medicine and Protection, Collaborative Innovation Center of Radiological Medicine of Jiangsu Higher Education Institutions, School of Radiation Medicine and Protection, Soochow University, Suzhou, China

**Keywords:** CYP24A1, 1α,25(OH)_2_D_3_, migration, invasion, epithelial–mesenchymal transition

## Abstract

Epithelial-mesenchymal transition (EMT) bestows cancer cells with motile and invasive properties. But for ovarian tissues, EMT plays a physiological role in the postovulatory repair of ovary surface epithelial (OSE) cells. Accumulating data indicated that 1α,25(OH)_2_D_3_ decreased both the migration and invasion of various cancer cells by suppressing EMT. However, it remains unclear whether 1α,25(OH)_2_D_3_ inhibits the process of EMT during different stages of oncogenic transformation in mouse OSE (MOSE) cells. In present study, a spontaneous malignant transformation model of MOSE cells at three sequential stages (early, intermediate and late) was established *in vitro* first and then subjected to 1α,25(OH)_2_D_3_ treatment to investigate the effect of 1α,25(OH)_2_D_3_ on the oncogenic transformation of MOSE cells. We found that 1α,25(OH)_2_D_3_ significantly reduced the proliferation and invasion of late malignant transformed MOSE (M-L cells) cells by inhibiting EMT both *in vitro* and *in vivo*, but not in intermediate transformed (M-I) cells. Importantly, we found that the levels of CYP24A1 in M-I cells were dramatically higher than that in M-L cells following treatment with 1α,25(OH)_2_D_3_. Furthermore, we demonstrated that, in both M-I and M-L cells with CYP24A1 knockdown, 1α,25(OH)_2_D_3_ suppressed the proliferation and invasion, and reduced the expression of N-cadherin, Vimentin, β-catenin and Snail. In addition, knockdown of CYP24A1 suppressed EMT by increasing E-cadherin while decreasing N-cadherin, Vimentin, β-catenin and Snail. These findings provide support for inhibiting CYP24A1 as a potential approach to activate the vitamin D pathway in the prevention and therapy of ovarian cancer.

## Introduction

Epithelial mesenchymal transition (EMT) is a multi-step process by which epithelial cells lose epithelial features and acquire characteristics of mesenchymal cells. The EMT is of great significance to processes involving loss of intercellular adhesion and cell polarity and acquisition of migratory and invasive capacities, such as embryonic development, wound healing, organ fibrosis, etc. Substantial evidence suggest that EMT is abnormally activated during the process of tumor initiation and development as well as in the acquisition of drug resistance (1). In ovarian tissue in particular, EMT plays not only a physiological role in the postovulatory repair of the ovary surface epithelial (OSE) cells, but also a role in the aggressiveness and recurrence of ovarian cancer (2). Ovarian epithelial carcinoma, accounting for more than 90% of all ovarian malignancies, is the most fatal gynecological malignancy due to its strong metastatic ability (3). Key events critical for the migration and invasiveness of epithelial ovarian cancer cells include the invasion of surrounding tissue, the generation of circulating cancer cells, intravasation and survival in the circulation system, the penetration of blood vessels, and the growth at secondary sites (4–6). These cells are endowed with invasion abilities and are able to initiate tumor generation at different sites by undergoing EMT (7). Thus, the development of new therapies able to suppress EMT is therefore needed.

In recent decades, 1α,25-dihydroxyvitamin D3 (1α,25(OH)_2_D_3_), which is the active form of vitamin D, has been investigated preclinically for its potential in anti-cancer therapy (8, 9). Suboptimal levels of 25 hydroxyvitamin D, the precursor of 1α,25(OH)_2_D_3_, are associated with a poor prognosis in patients with ovarian cancer (9). In addition, results have indicated that 1α,25(OH)_2_D_3_ and its synthetic derivatives inhibit metastasis of many kinds of cancers, including colon, pancreatic, and breast cancers by impeding the progress of an EMT (10–12). We also found that 1α,25(OH)_2_D_3_ suppresses the migration of human ovarian cancer cells by inhibiting EMT (13). However, a meta-analysis did not identify a significant correlation between serum 25, hydroxyvitamin D levels and the risk of ovarian cancer (16), and the efficacy of 1α,25(OH)_2_D_3_ on ovarian cancer has yet to be supported by results from randomized controlled trials (14). Thus, it is important to investigate the mechanisms underlying the anti-cancer effects of 1α,25(OH)_2_D_3_.

CYP24A1 is a member of the cytochrome P450 family, the main function of which is to convert 25 hydroxyvitamin D and 1α,25(OH)_2_D_3_ to 24,25 hydroxyvitamin D and 1α,24,25(OH)_2_D_3_, the inactive form of vitamin D (8). This process is a physiological protection to avoid the hypercalcemia that can be induced by high levels of 1α,25(OH)_2_D_3._ It has been demonstrated that the expression of CYP24A1 is increased in prostate cancer and ovarian cancer cells after treatment with 1α,25(OH)_2_D_3_ *in vitro* (15, 16). Other studies have shown that increasing levels of CYP24A1 reverse the anti-cancer effects of 1α,25(OH)_2_D_3_ (17– 19). Therefore, further investigation is needed to examine whether reduced CYP24A1 levels could enhance the anti-tumor efficacy of 1α,25(OH)_2_D_3_ *via* suppressing EMT.

We have successfully built a spontaneous malignant transformation model of mouse OSE (MOSE) cells, and found that EMT was associated with the oncogenic transformation of MOSE cells. In the present study, We aimed to investigate whether 1α,25(OH)_2_D_3_ could suppress the migration and invasion of MOSE cells during malignant transformation, and whether knockdown of CYP24A1 enhances the anti-cancer effects of 1α,25(OH)_2_D_3_ by regulating EMT.

## Materials and Methods

### MOSE Cell Isolation and Culture

MOSE cells were isolated using the method described by Roby et al. (20), and the model of spontaneous malignant transformation was established by our group (20). Briefly, single MOSE cells were collected from the ovaries of female C57BL/6 mice. Next, cells were cultured in Dulbecco's Modified Eagle's Medium/Nutrient Mixture F-12 (DMEM/F12, Invitrogen, Carlsbad, CA) culture media, supplemented with 20 ng/ml mEGF, 20 ng/ml mouse basic FGF, 2 μg/ml insulin and 4 μg/ml heparin sodium (Gibco, USA) in a humidified atmosphere of 5% CO_2_ at 37°C. MOSE cells were continuously passaged *in vitro*. Over time, MOSE cells underwent spontaneous neoplastic transformation when subcultured for more than 80 passages *in vitro*. Based on morphological changes, chromosomal number, and proliferation ability, three sequential stages of transformed MOSE cells were defined as follows (data not published): early passage MOSE cells (M-E, ≤ 20 passages), intermediate-passage MOSE cells (M-I, 21–79 passages), and late-passage MOSE cells (M-L, ≥ 80 passages). The MOSE cells have been tested for mycoplasma and human cell lines contamination by STR profiling and were demonstrated to be clean (21). We did test the cytotoxicity of 1α,25(OH)_2_D_3_ at doses of 1, 10, and 100 nM in three sequential stages of transformed MOSE cells, and found no significant inhibitory effects of 1α,25(OH)_2_D_3_ on cell viability at the dose of 1 nM (Supplementary Figure 1). In this study, M-I and M-L cells were continuously treated with 1 nmol/L 1α,25(OH)_2_D_3_ (Sigma, St. Louis, MO, USA).

### Colony Formation and Soft Agar Colony Formation Assay

According to the previous protocol by Sun et al. (23), one thousands cells were seeded into a 6-well plates. The cells were cultured in a humidified incubator at 37°C with 5% CO_2_ for 2 weeks. The medium was changed for every 3 days. The cell colonies were fixed with methanol for 15 min and stained with Giemsa's solution for 15 min, then washed with water and air-dried. The colonies with the diameter >0.5 mm were identified with ImageJ software. Plate colony formation efficiency was estimated by the number of clones divided by the number of cells inoculated. All experiments were repeated for 3 times and the average value was presented.

Cell suspensions were prepared as described above. A base layer of the mixture of 2 × Ham's F-12 (Sigma Aldrich, 1/2) including 20% fetal bovine serum, and ultrapure LMP agarose (Sigma Aldrich, 1/2) was dissolved at 56°C for 20 min and then warmed to 37°C before the addition of top layer. A total of 2.5 × 10^4^ cells were resuspended in 2 × Ham's F-12 medium, and ultrapure LMP agarose, (1:1) and gently added onto the base layer. The cells were incubated at 37°C for 14 days, and 1 ml of fresh medium was added to the top layer every 3 days. Colonies were visualized using the EVOS XL imaging system (Life Technologies) and counted using ImageJ software.

### Migration and Invasion Assays

The migration of MOSE cells was assessed using a wound healing assay. Cells were plated into a 6-well plate with FBS-free media for 12 h. Afterwards, cells cultured in the bottom of the well were scratched using a pipette tip to create a wound area. After 24 h, wounds (3 images each well) were imaged under a microscope (×40, CKX41F, Olympus, Tokyo, Japan) to detect the width of the gaps. Wound healing assay data is displayed as the migration index (%) = [(initial width)–(final width)]/(initial width). Values were normalized to the control group. Data points in the figure represent three independent experiments. For the detection of invasiveness, cells were seeded into Boyden Matrigel-coated chambers with 8 μm pore inserts (BD Bioscience, San Jose, CA, USA). The lower chamber was supplemented with DMEM/ F12 media containing 10% FBS. After 24 h, the non-invasive cells were cleaned using a cotton swab. Then, cells at the bottom of the chamber were stained with crystal violet (c0121, Beyotime Biotechnology, Shanghai, China), and imaged under a microscope (×400, CKX41F, Olympus, Tokyo, Japan). The stained cells were then dissolved in 0.5 ml ethanol. Finally, OD values were obtained at 595 nm using a microplate reader (Molecular Devices, FiltorMax F5, USA).

### Animal Experiments

Four to six weeks old (18–20 g) female BALB/C nude mice were purchased from the Shanghai Laboratory Animal Centre, Chinese Academy of Sciences, China. Animals were kept in a specific pathogen-free environment, with a temperature of 21 ± 2°C, relative humidity of 55 ± 5% and a 12:12 h light-dark cycle. Mice were provided with water and food *ad libitum*. All surgical procedures and care provided to the animals were approved by the Institutional Animal Care and Use Committee of Soochow University (approval number is ECSU 201800049). A 200 μl cell suspension containing 10^5^ MOSE cells were injected intraperitoneally into mice. Ten mice were randomly assigned to vehicle (sesame oil with 2% ethanol) group (*N* = 5), and vitamin D3 (1,000 IU/week) treatment group (*N* = 5). Treatments were administered for 8 weeks. Body weight was monitored weekly. All mice were euthanized by cervical dislocation prior to tissue collection. The tumors were then dissected out and weighed. Tumor tissue was then fixed in formalin for 1 week. The tumors were dehydrated and cut into 4 μm sections of paraffin-embedded tissue samples. The slices were then stained with hematoxylin and eosin (H&E).

### Bioinformatic Data Analysis of GSE26903

GSE26903 data were retrieved from the National Center for Biotechnology Information's Gene Expression Omnibus (GEO) (https://www.ncbi.nlm.nih.gov/geo/query/acc.cgi?acc=GSE26903), which was microarray analysis result of gene expressions in 1,25-dihydroxyvitamin D3-treated ovarian cancer OVCAR3 cells. Raw data extraction and analysis were performed by the R software package (version 2.15, http://www.r-project.org/). For GSE26903, the edger package was used for DEG screening. *P*-value <0.05 and absolute log_2_FC bigger than 1 were used as the cut-off criteria based on Benjamini & Hochberg (BH) procedure. Intersect function in R was used for identifying DEGs. The Venn diagram was generated by VennDiagram R package.

### Western Blotting

MOSE cells were lysed using RIPA buffer (P0013, Beyotime Biotechnology, Shanghai, China), and a BCA protein analysis kit (P0012, Beyotime Biotechnology, Shanghai, China) was used to determine protein concentration. Samples (20 μg protein per sample) were then separated by 10% SDS-PAGE (P0012A, Beyotime Biotechnology, Shanghai, China), followed by transfer to a polyvinylidene fluoride (PVDF) membrane (Millipore, Boston, MA, USA). Membranes were blocked with 5% skim milk and incubated overnight with primary antibodies at 4°C. Then, membranes were rinsed in Tween-20 phosphate buffer (PBST) three times, followed by incubation with secondary antibodies for 1 h at room temperature. Afterwards, membranes were washed three times in PBST. A chemiluminescent reagent (Millipore, Boston, MA, USA) was used to visualize the protein complexes. ImageJ was used to read the grayscale values (Integrated Optical Density, IOD). The relative expression level of targeted proteins relative to β-actin (the loading control) was then quantified. Data in figures represent at least three independent experiments. The following antibodies were acquired from Cell Signaling Technology (Irvine, CA, USA): E-cadherin (1:700, #5296), Vimentin (1:800, #5741), β-catenin (1:100, #9582), Snail (1:700, #3879), N-cadherin (1:700, #4061), β-actin (1:1,000, #3700), HRP-linked anti-rabbit IgG (1:2,000, #7074), HRP-linked anti-mouse IgG (1:2,000, #7076). CYP24A1 (1:500, ab203308) primary antibody was purchased from Abcam (Cambridge, MA, USA).

### Immunofluorescence Staining

Twenty thousands MOSE cells were cultured on a glass cover slip (Thermo Fisher Scientific, Waltham, MA, USA), which was placed into 24-well plates. MOSE cells were treated with 1α,25(OH)_2_D_3_ after cells had adhered to the cover slips. To prepare for immunostaining, cells were rinsed twice with fresh PBS, followed by fixing in 4% paraformaldehyde for 20 min. Then, 0.1% Triton was used to permeabilize cells at 4°C for 15 min, and nonspecific binding was blocked with 5% skim milk for 1 h at room temperature. Cells were then incubated with the following primary antibodies: E-cadherin (1:100), Snail (1:50) and β-catenin (1:100). Cells were then incubated with secondary antibody IgG-Cy5 (1:1,000, #4412, Cell Signaling Technology) for 1.5 h. Cell nuclei were labeled with DAPI. A confocal laser scanning microscope (TCS SP2, Leica, Wetxlar, Germany) was used to obtain fluorescent images.

### RT-qPCR

Total RNA from MOSE cells was extracted with an RNeasy kit (Qiagen, Crawley, West Sussex, UK). The Transcriptor First Strand cDNA Synthesis Kit (Roche, Mannheim, Germany) was then used to perform reverse transcription with 1 μg total RNA. Quantitative RT-PCR was performed to test the levels of CYP24A1 in MOSE cells and ascites cells, which were collected from mice injected with MOSE cells. The CYP24A1 primer set sequences were as follows: forward: 5′-CTGCCCCATTGACAAAAGGC−3′; reverse: 5′- CTCACCGTCGGTCATCAGC−3′. The β-actin primer set sequences were as follows: forward: 5′- GGCTGTATTCCCCTCCATCG−3′; reverse: 5′- CCAGTTGGTAACAATGCCATGT−3′. The SYBR-Green I Nucleic Acid Gel stain (Roche) was applied to the qPCR. An ABI 7500 real-time PCR system was used to perform the RT-PCR reactions (Applied Biosystems, Foster City, CA, USA). Thermocycling parameters were: 95°C for 10 min, followed by 40 cycles of 95°C for 10 s, 60°C for 20 s and 72°C for 30 s. 2^−ΔΔCq^ method was used to calculated the relative expression levels (22). Six replicates for each sample were used for the calculations in each of the three independent experiments. The β-actin was used as an internal control.

### Specific Lentiviral Knockdown of CYP24A1

CYP24A1 knockdown cells were produced by lentivirus infection. First, an online shRNA design tool MISSION® from Sigma-Aldrich was used to determine a set of top-scoring targets for CYP24A1. Then, shRNA oligo (5′-CCGGGCACAATTTCATAAACGCAAACTCGAGTTTGCG TTTATGAAATTGTGCTTTTTTG-3′), named as sh-CYP24A1, was synthesized for cloning into a PLKO.1 backbone. PLKO.1-scramble shRNA targeting no known genes from any species was used as a negative control, named as sh-NC (Sigma-Aldrich, SHC016). We then co-transfected psPAX2 and PLKO.1-shRNA plasmids into 293T cells. The pMD2.G plasmid was then enveloped and used to generate the sh-CYP24A1 lentivirus. The supernatant of 293T cells was collected after 48 h, and filtered using a 0.45 μm membrane. A mixture of lentivirus and media was combined with 5 ml media containing 8 mg/ml polybrene. MOSE cells were treated for 24 h with lentivirus-containing mixture followed by selection using puromycin (1 mg/ml) for an additional 48 h. Cells were then cultured for further analysis.

### Statistical Analysis

Statistical analyses were performed using GraphPad Prism 7 (GraphPad Software Inc., La Jolla, CA, USA). Quantitative data are presented as the mean ± standard deviation of the mean (SD). Statistical data were analyzed using a two-tailed, unpaired Student's *t*-test. *P* < 0.05 was considered statistically significant.

## Results

### 1α,25(OH)_2_D_3_ Partially Suppresses the Proliferation and Invasion of MOSE Cells Both *in vitro* and *in vivo*

**Figure 1:**
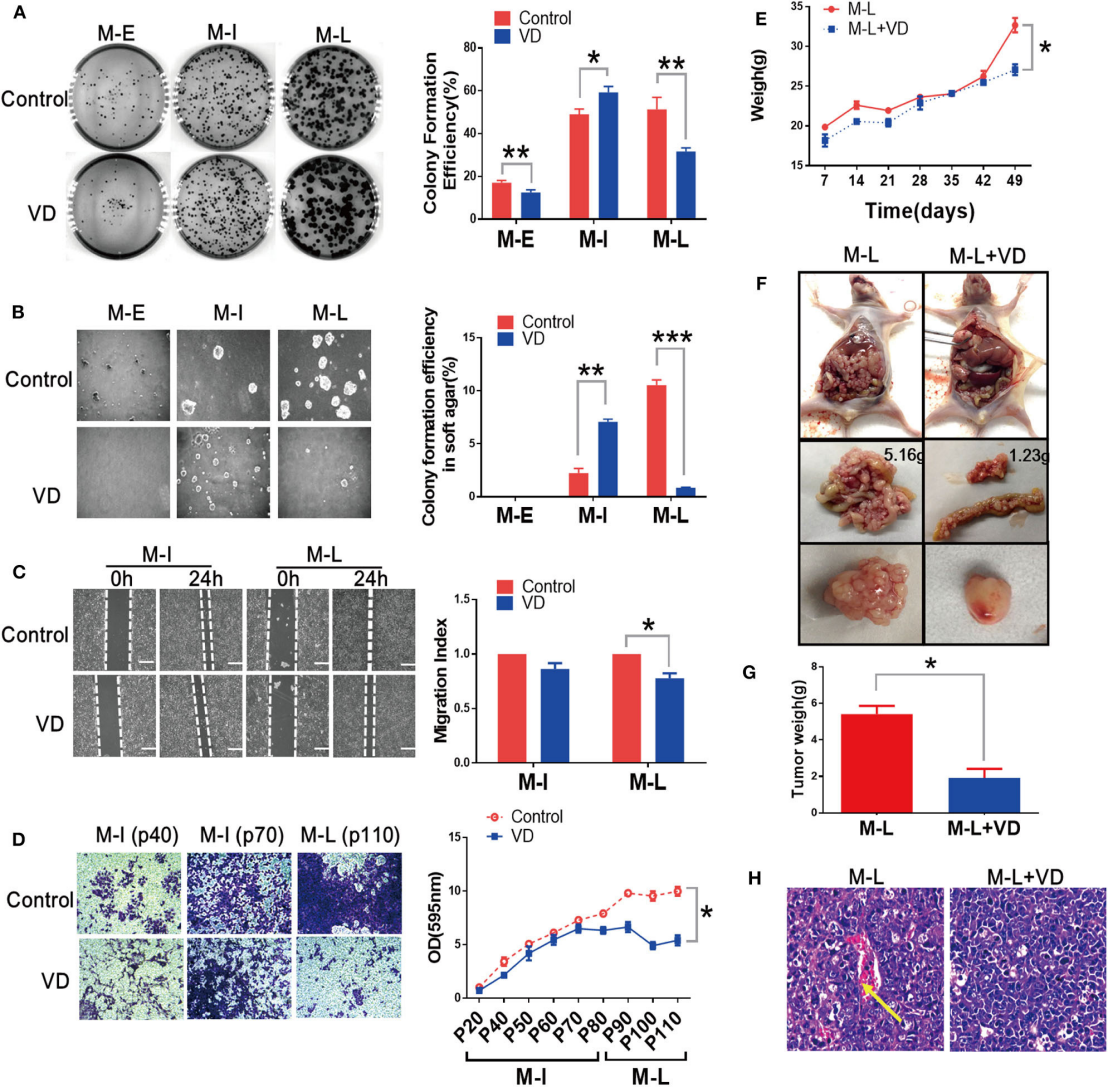
1α,25(OH)_2_D_3_ partially suppresses the proliferation and invasion and migration of MOSE cells both *in vitro* and *in vivo*. **(A)** Left: Representative images of colony formation after 14 days. Right: Colony formation efficiency at different stages of MOSE cells with or without 1,α25(OH)_2_D_3_ treatment. Colonies were counted using Image J software. **(B)** Left: Representative images of colonies in soft agar. Colonies were visualized after 7 days using the EVOS XL imaging system (magnification, 20×). Right: Effect of 1,α25(OH)_2_D_3_ on colony formation efficiency in soft agar at different stages of MOSE cells. **(C)** Left: the migration of M-I and M-L cells was detected using a wound healing assay. Images were taken after the wound was created (0 h) and 24 h later. Right: the migration index was calculated as follows: migration index = [(the initial width of the scratch)–(the final width of the scratch)]/(the initial width of the scratch). The values were normalized to the control group. **(D)** Left: the invasion of M-I and M-L cells was determined using a transwell assay. A transwell-invasion assay was performed using 24-well Boyden Matrigel-uncoated chambers (magnification, 400×). Right: the cells on the bottom of inserts were dissolved using ethanol. Then, the OD value of the solution was measured. **(E)** Mouse body weight was measured every week after injection. **(F)** Tumors were dissected from mice and representative images of tumors from vehicle or 1α,25(OH)_2_D_3_-treated M-L cells were shown. **(G)** Tumor weight was measured after dissecting. **(H)** H&E staining of tumor tissues showed the pathological features in control and 1α,25(OH)_2_D_3_-treated groups (100× magnification). The yellow arrow indicates blood vessels and cells with an irregular shape. Data are expressed as the mean ± standard deviation (SD). *p < 0.05, **p < 0.01, and ***p < 0.001. Data points in figures represent three independent experiments.

Previous study from our group has revealed that 1α,25(OH)_2_D_3_ represses the migration of human ovarian cancer SKOV-3 cells (13). We wondered whether 1α,25(OH)_2_D_3_ could hold back malignant transformation of MOSE cells. The MOSE cells were continually treated with 1,α25(OH)_2_D_3_ from early (M-E), intermediate (M-I) to late (M-L) passages. The colony formation efficiency in 1,α25(OH)_2_D_3_-treated M-E cells (12.467 ± 1.286%) was significantly lower than that of untreated M-E cells (17.133 ± 1.026%) (*p* < 0.01). And, 1,α25(OH)_2_D_3_ significantly inhibited colony formation efficiency in M-L cells (31.600 ± 1.778%) compared with untreated M-L cells (51.333 ± 5.630%). However, 1,25(OH)_2_D_3_-treated M-I cells exhibited significantly higher colony formation efficiency than the untreated cells (*p* < 0.05) (Figure 1A). Meanwhile, we observed that M-I and M-L, but not M-E cells, could form colonies in soft agar. 1,α25(OH)_2_D_3_-treatment significantly increased the colony formation efficiency of M-I cells in soft agar (*p* < 0.01), but it dramatically inhibited the colony formation efficiency of M-L cells (*p* < 0.01) (Figure 1B). As regard to migration, we found that there was no obvious difference in the migration index between the 1α,25(OH)_2_D_3_-treated group and the control group among M-I cells. However, 1α,25(OH)_2_D_3_ significantly inhibited the migration of M-L cells compared with untreated cells (*p* < 0.05) (Figure 1C). Similarly, 1α,25(OH)_2_D_3_ significantly decreased the invasion abilities of M-L cells, but it did not work in M-I cells (*p*<0.05; Figure 1D). These results indicated that 1,α25(OH)_2_D_3_ suppressed the proliferation and invasion of both M-E and M-L cells, but not in M-I cells.

Next, we determined the metastatic abilities of MOSE cells treated with or without 1α,25(OH)_2_D_3_ *in vivo*. 1α,25(OH)_2_D_3_-treated or control MOSE cells were injected intraperitoneally into nude mice. We found that mice injected with M-I cells failed to form tumors, those injected with M-L cells produced tumors. As shown in Figure 1E, the body weights of mice injected with 1α,25(OH)_2_D_3_-treated M-L cells were significantly lower than the control group (*p* < 0.05). 1α,25(OH)_2_D_3_ suppressed the metastasis of M-L cells compared to untreated-M-L cells *in vivo* (1.2335 vs. 5.1561 g). The ovarian tumors formed from 1α,25(OH)_2_D_3_-treated M-L cells were smaller than those from control M-L cells (*p* < 0.05, Figures 1F,G). H&E staining of tumor tissues showed that there were fewer irregularly shaped blood vessels and cells in the 1α,25(OH)_2_D_3_-treated group than in the corresponding control group (Figure 1H). These findings implied that 1α,25(OH)_2_D_3_ dramatically inhibits the metastasis of M-L cells, yet has no effect on the M-I cells.

### 1α,25(OH)_2_D_3_ Inhibits EMT During Malignant Transformation of MOSE Cells

**Figure 2:**
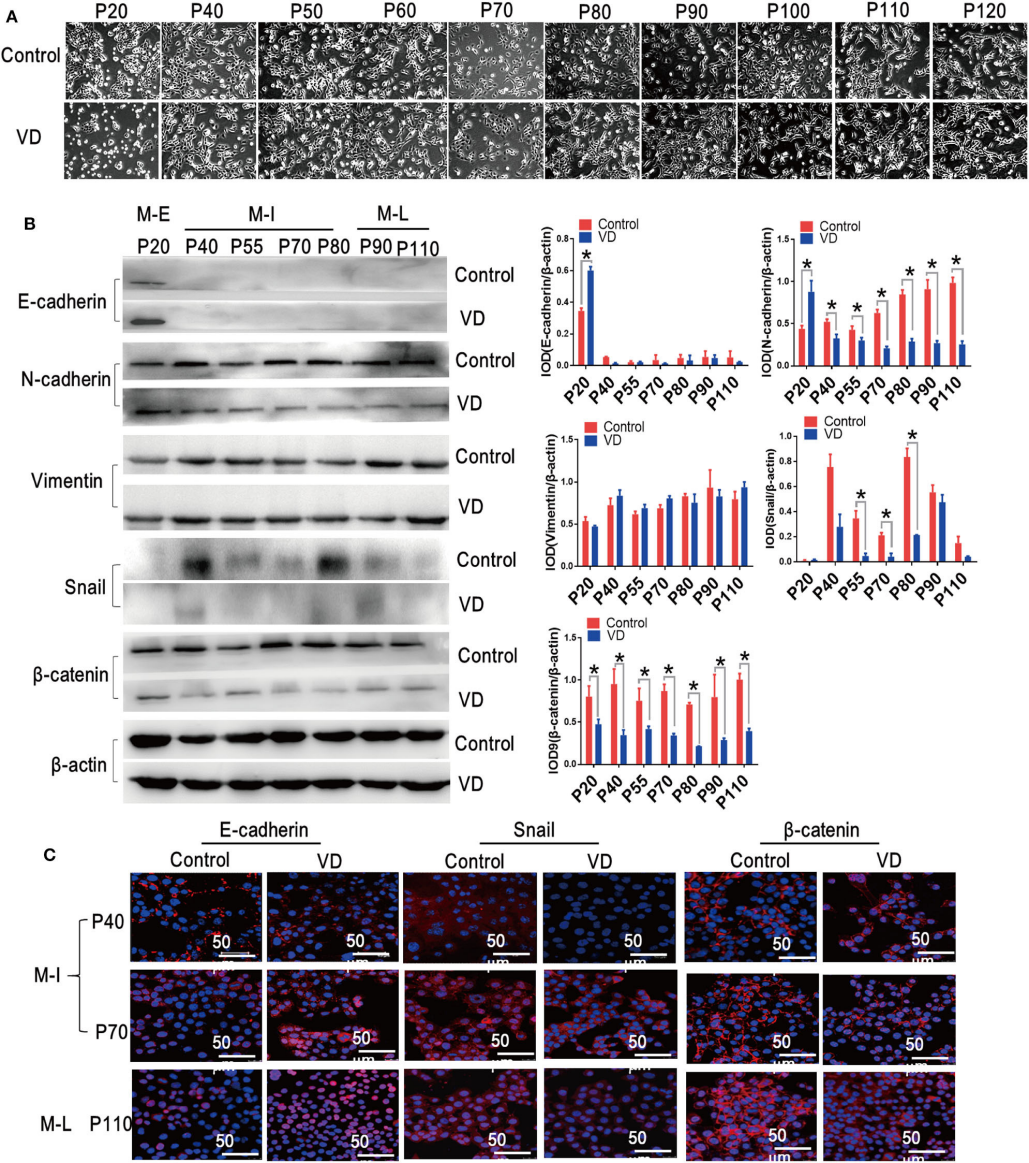
1α,25(OH)_2_D_3_ inhibits EMT during the spontaneous malignant transformation of ovarian epithelial cells. **(A)** Cellular morphology images were taken during the malignant transformation of MOSE cells treated with 1α,25(OH)_2_D_3_ (magnification, 400×). **(B)** The protein levels of E-cadherin, N-cadherin, Vimentin, Snail and β-catenin as determined by western blotting. ββ-actin served as a loading control. The level of the indicated protein was quantified with gray value (Integrated Optical Density, IOD). Data were expressed as mean ± SD. * means *p* < 0.05. Data points in figures represent three independent experiments. **(C)** Representative confocal laser scanning microscope images of the indicated proteins. Red color represents E-cadherin, Snail and β-catenin, respectively. Nuclear DNA was visualized using DAPI staining (magnification, 200×).

EMT plays an essential role in transforming epithelial cells into mesenchymal cells with more aggressive phenotype (23). Interestingly, we also observed that MOSE cells acquired a spindle-like shape during spontaneous malignant transformation, but this morphological change was blocked by 1α,25(OH)_2_D_3_ (Figure 2A). To investigate whether 1α,25(OH)_2_D_3_ inhibits EMT during the spontaneous malignant transformation of MOSE cells, we thus examined a series of EMT markers and transcription factors. We found that 1α,25(OH)_2_D_3_ decreased the expression of N-cadherin in both M-I and M-L cells, compared with control cells (*p* < 0.05; Figure 2B). However, 1α,25(OH)_2_D_3_ did not affect the expression levels of E-cadherin, except in early cells (p20). Furthermore, 1α,25(OH)_2_D_3_ significantly decreased the levels of Snail in M-I cells, and β-catenin in M-I and M-L cells (Figure 2B). Immunofluorescence staining also revealed similar results, with the exception of E-cadherin (Figure 2C). Together, results from the morphological phenotype, invasion ability, and protein expression patterns indicated that 1α,25(OH)_2_D_3_ partially inhibited the EMT process.

### Long-Term Treatment With 1α,25(OH)_2_D_3_ Results in Elevated Levels of CYP24A1

**Figure 3:**
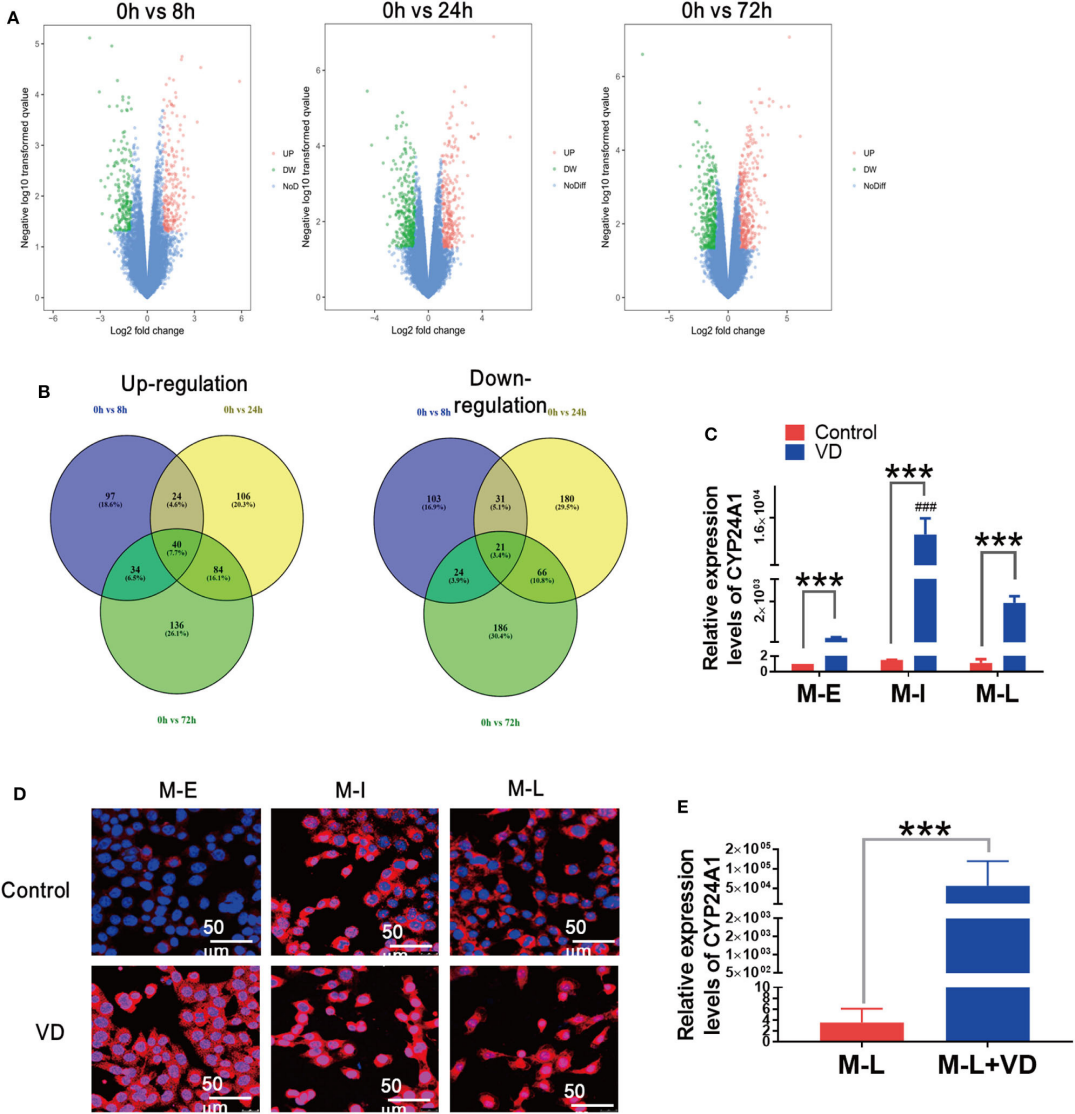
Long-term administration of 1α,25(OH)_2_D_3_ results in a significant rise in CYP24A1 and delays the alteration of cellular morphology. **(A)** Volcano plots of DEGs in 1,α25(OH)_2_D_3_ treated-OVCAR3 cells for 8, 24, and 72 h. X-axis indicates the fold change (log scale), whereas Y-axis shows the *p*-values (log scale). Each symbol represents a differentially expressed gene, and the symbols in red/green categorize the up-/down-regulated genes falling under different criteria (*p*-value and fold change threshold). *p* <0.05 is considered as statistically significant, whereas absolute log_2_FC > 1 is set as the threshold. **(B)** The overlapped up-/down-regulated DEGs in 1,α25(OH)_2_D_3_-treated OVCAR3 cells for 8, 24, and 72 h. **(C)** The mRNA levels of CYP24A1 in control and 1α,25(OH)_2_D_3_-treated MOSE cells of different stages. **(D)** The expression and distribution of CYP24A1 in control and 1α,25(OH)_2_D_3_-treated MOSE cells of different stages. **(E)** The mRNA levels of CYP24A1 in ascites cells collected from mice injected with vehicle or 1α,25(OH)_2_D_3_-treated M-L cells. Data are expressed as the mean ± SD. ***p < 0.001. Data points in figures represent three independent experiments. ###*p* < 0.001 when compared with VD-treated M-E or M-L cells.

To identify differentially expressed genes (DEGs) regulated by 1,α25(OH)_2_D_3_ in inhibiting proliferation and invasion of ovarian cancer cells, we searched the microarray data of human ovarian adenocarcinoma OVCAR3 cells by the National Center for Biotechnology Information's Gene Expression Omnibus (GEO). The full set of raw data from this study was accessible from the link: https://www.ncbi.nlm.nih.gov/geo/query/acc.cgi?acc=GSE26903. Volcano plots were generated to visualize the distribution of DEGs between untreated and 1,α25(OH)_2_D_3_-treated OVCAR3 cells at 8, 24, and 72 h. Red or green dots in the plots represented significantly up-regulated or down-regulated genes, respectively (Figure 3A). Based on absolute log_2_FC > 1 cut-off criteria, total 61 were differentially expressed in common among three groups (*p* < 0.05). Among them, 40 genes were up-regulated and 21 were down-regulated (Figure 3B). In 1,α25(OH)_2_D_3_-treated OVCAR cells, CYP24A1 was the most up-regulated genes by 58-, 67-, and 69-fold increase after 8, 24, and 72 h treatment, respectively.

Rising levels of CYP24A1 induced by 1α,25(OH)_2_D_3_ can antagonize the anti-metastatic effects of 1α,25(OH)_2_D_3_ (16). To investigate whether the partial anti-metastatic effects of 1α,25(OH)_2_D_3_ were associated with rising levels of CYP24A1, we measured CYP24A1 levels in M-E, M-I and M-L cells using qRT-RCR. The enhancements of CYP24A1 in M-I cells (>12,000-fold) was more obvious than M-E cells (370-fold) and M-L cells (1,600-fold) (*p* < 0.0001) (Figure 3C). The result from immunofluorescence staining also showed that the expression of CYP24A1 in M-I cells was more increased than that of other two cell lines. Additionally, CYP24A1 was diffusely located in the nuclei of M-I cells, but mainly expressed in the cytoplasm of M-E and M-L cells. On the contrary, CYP24A1 expression exhibited an opposite trend in 1,α25(OH)_2_D_3_-treated cells (Figure 3D). Similarly, there was a drastic increase in CYP24A1 expression (>15,000-fold) in ascites cells collected from mice injected with 1α,25(OH)_2_D_3_-treated M-L cells (Figure 3E). These findings demonstrate that the enhancement of CYP24A1 in M-I cells is higher than in M-L cells following 1α,25(OH)_2_D_3_ treatment, which may be caused by the antagonism of CYP24A1 by 1α,25(OH)_2_D_3_.

### Knockdown of CYP24A1 Augments the Anti-Invasion Effects of 1α,25(OH)_2_D_3_

**Figure 4:**
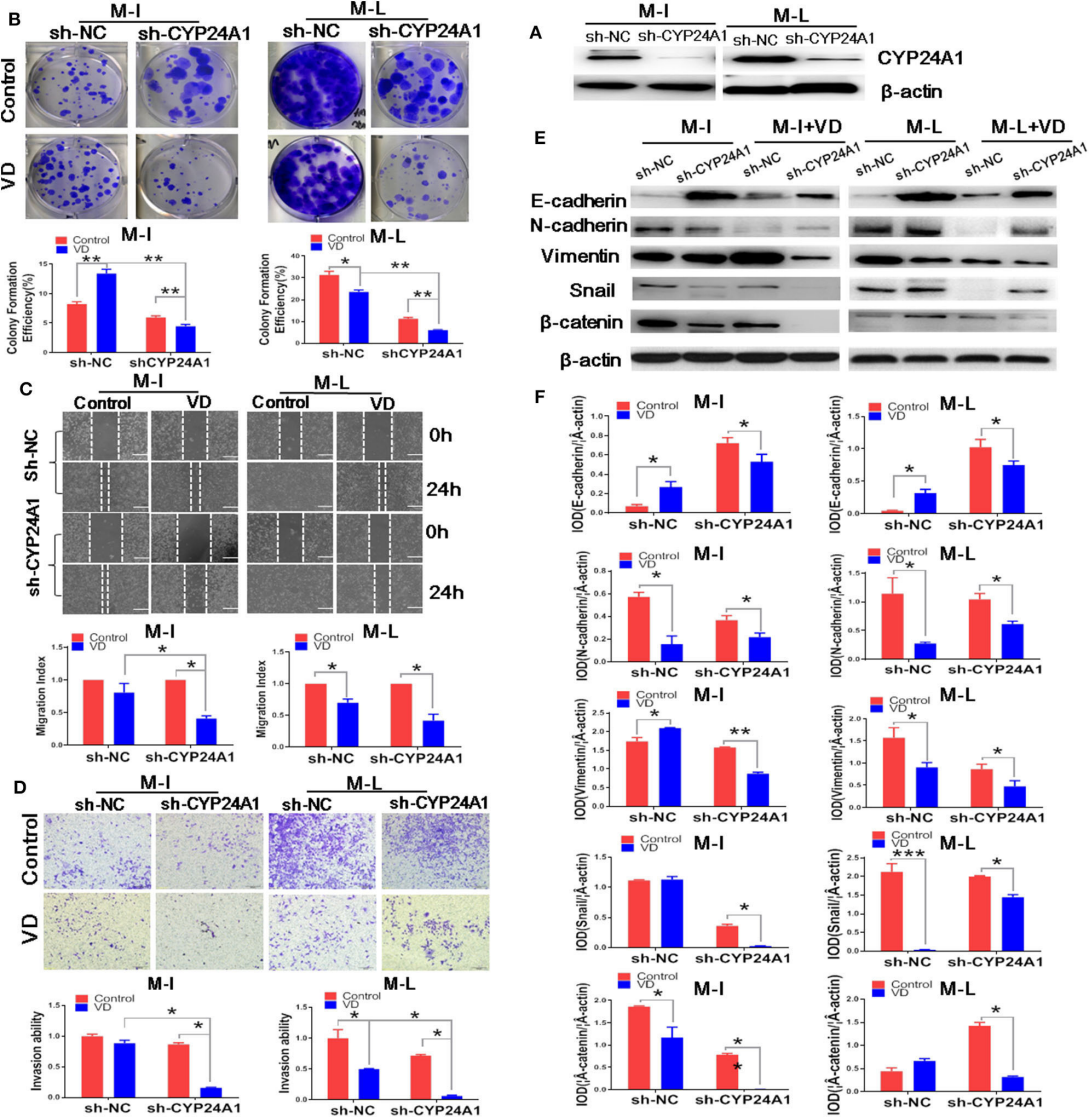
Knockdown of CYP24A1 enhances the anti-metastasis and EMT-inhibiting effects of 1α,25(OH)_2_D_3_ in MOSE cells. **(A)** Stable knockdown of CYP24A1 in M-I and M-L cells was performed using PLKO.1 system. **(B)** Effect of CYP24A1 knockdown on colony formation efficiency in 1,a25(OH)_2_D_3_-treated M-I and M-L cells. The colonies were counted using Image J software. Representative images of colony formation were shown and the photographs were taken 14 days after 1,a25(OH)_2_D_3_ treatment. **(C)** Upper panel: the migration of M-I and M-L cells following CYP24A1 knockdown, as determined by a wound healing assay. Images were taken after the wound was created (0 h), and 24 h later. Lower panel: the migration index was calculated as follows: migration index (%) = [(the initial width of the scratch)–(the final width of the scratch)]/(the initial width of the scratch). Values were normalized to the control group. Data are expressed as the mean ± SD. *p < 0.05. **(D)** Upper panel: the invasion of M-I and M-L cells following CYP24A1 knockdown, as detected using a transwell assay. Lower panel: cells that were adhered to the bottom of inserts were counted under a microscope. Invasion ability was calculated as follows: invasion ability = (the number of cells on the bottom of inserts)/(the vector control cells on the bottom of inserts). Data are expressed as the mean ± SD. *p < 0.05. **(E,F)** The expression levels of EMT-related proteins (E-cadherin, N-cadherin, Vimentin, β-catenin and Snail) were detected by western blotting in empty vector-transfected or CYP24A1-knockdown (sh-CYP24A1) MOSE cells. The levels of the indicated proteins were quantified with gray value (Integrated Optical Density, IOD). Data were expressed as mean ± SD. *p < 0.05 and **p < 0.01. Data points in figures represent three independent experiments.

Then, we sought to determine whether knockdown of CYP24A1 could enhance the anti-invasion properties of 1α,25(OH)_2_D_3_. Figure 4A demonstrates the stable knockdown of CYP24A1 in M-I and M-L cells using the PLKO.1 lentiviral system. Although 1,α25(OH)_2_D_3_ promoted the growth of sh-NC M-I cells, the knockdown of CYP24A1 meaningfully inhibited colony formation efficiency of both M-I and M-L cells treated by 1,α25(OH)_2_D_3_ (Figure 4B), indicating that silencing CYP24A1 enhanced the effect of 1,α25(OH)_2_D_3_ on inhibiting growth of MOSE cells. As demonstrated in Figure 4C, 1α,25(OH)_2_D_3_ did not inhibit the migration of M-I cells transfected with sh-NC, but strongly inhibited the migration of sh-CYP24A1-transfected M-I cells. And it markedly reduced the migration of both NC and CYP24A1 knockdown M-L cells. Moreover, we found that the migration index in sh-CYP24A1-transfected M-I cells was lower than the sh-NC group, following treatment with 1α,25(OH)_2_D_3_ (*p* < 0.05). Similarly, the invasion ability of CYP24A1 knockdown cells was weaker than that of sh-NC cells, in both M-I and M-L cells treated with 1α,25(OH)_2_D_3_ (*p* < 0.05; Figure 4D). Subsequently, results from western blotting showed that only knockdown of CYP24A1 elevated the levels of E-cadherin in both M-I and M-L cells, not concerning 1α,25(OH)_2_D_3_. 1α,25(OH)_2_D_3_ increased the E-cadherin in sh-NC cells, but decreased that in shCYP24A1-transfected M-I and M-L cells. Further, 1α,25(OH)_2_D_3_ reduced the expressions of N-cadherin, Vimentin, Snail and β-catenin significantly in both M-I and M-L cells with shCYP24A1 knockdown (*p* < 0.05, Figures 4E,F). These results further demonstrated that the loss of CYP24A1 increased the anti-invasion properties of 1α,25(OH)_2_D_3_ by suppressing EMT.

## Discussion

Some evidence indicated that CYP24A1 overexpression resulted in cellular resistance to the antiproliferative effects of 1α,25(OH)_2_D_3_ (8, 17, 24). The high levels of CYP24A1 also positively correlated with a poor outcome of cancers including ovarian, prostate, breast, thyroid, colorectal cancers and melanomas (14–18, 21). In this study, we found that 1α,25(OH)_2_D_3_ significantly reduced the migration and invasion of malignant MOSE (M-L) cells by inhibiting EMT, yet had no effect on intermediate MOSE (M-I) cells. Interestingly, the levels of CYP24A1 in M-I cells were distinctly higher than in M-L cells following continuous treatment with 1α,25(OH)_2_D_3_. Furthermore, we demonstrated that loss of CYP24A1 enhanced the anti-invasion properties of 1α,25(OH)_2_D_3_ by suppressing EMT. These findings also imply that 1α,25(OH)_2_D_3_ in combination with CYP24A1 inhibitor may have potential in the treatment of ovarian cancer.

EMT is a process which can endow epithelial cells with increased capacity for invasiveness and mobility, giving them properties of mesenchymal cells (23). In most cases, EMT plays not only an essential role in embryonic development but also serves an important biological function in normal OSE cells. The EMT of OSE cells in response to postovulatory stimuli has been thought to enhance their postovulatory repair (25). However, abnormalities in the EMT process resulted in the uncontrolled proliferation of OSE cells, which may lead to tumorigenesis (26). Moreover, many reports have shown that EMT is relevant to dissemination and metastasis of ovarian cancer (27–29).

There are several different ways by which 1α,25(OH)_2_D_3_ exerts anti-tumor effects, including the induction of cellular apoptosis and differentiation, and the inhibition of proliferation and angiogenesis (9). Moreover, 1α,25(OH)_2_D_3_ has been shown to repress the migration and invasion abilities of multiple types of cancer cells, including prostate cancer (30), breast cancer (31), and pancreatic cancer (32). 1α,25(OH)_2_D_3_ and its synthetic derivatives also have anti-cancer effects in animal models (10, 33, 34). Briefly, the results of some studies have indicated that 1α,25(OH)_2_D_3_ decreases the migration and invasion of malignant tumor cells by suppressing EMT (10–13, 35). It has also been reported that 1α,25(OH)_2_D_3_ suppresses the migration of ovarian cancer (12), lung cancer (35) and colon cancer cells (10) through repressing TGF-β1-induced EMT. In this study, we found that 1α,25(OH)_2_D_3_ represses EMT by inhibiting β-catenin, N-cadherin and Snail expressions in malignant M-L cells. However, 1α,25(OH)_2_D_3_ showed no effect on E-cadherin in M-I or M-L cells, and it reduced the E-cadherin expressions when CYP24A1 was knocked-down. The recent study revealed that E-cadherin functions as a survival factor in invasive breast cancer during nearly all phases of metastasis, which hints strategy to inhibit E-cadherin-mediated survival may have potential as a therapeutic approach (36). Meanwhile, another research reported that 1α,25(OH)_2_D_3_ is efficient in countering EMT phenotype only when combined with TGF-β, but not at a later stage (37). Thus, it is worth to explore why 1α,25(OH)_2_D_3_ decreased the of E-cadherin expression while it inhibited the invasion of ovarian cancer cells.

Although 1α,25(OH)_2_D_3_ reduced the expression of N-cadherin, Snail and β-catenin in intermediate transformation (M-I) cells, it was unable to decrease their migration and invasion properties. We found that the expression of CYP24A1 in M-I cells was dramatically higher than that in M-L cells following 1α,25(OH)_2_D_3_ treatment. Previous research has also revealed aberrantly high levels of CYP24A1 in multiple kinds of cancer cells, including ovarian cancer, breast cancer, thyroid cancer, and glioma cells (38–41), thereby making them resistant to 1α,25(OH)_2_D_3_. Loss of CYP24A1 therefore facilitated the anti-tumor efficacy of 1α,25(OH)_2_D_3_ in lung (42), colorectal (19), endometrial (43) and thyroid cancer cells (17). These studies further revealed that knockdown of CYP24A1 repressed the Wnt/β-catenin and Akt signaling pathways. In the present study, we found that 1α,25(OH)_2_D_3_ only restricted the migration and invasion abilities of M-L cells but not M-I cells, which may be owing to the higher CYP24A1 expression levels in M-I cells. After knockdown of CYP24A1, 1α,25(OH)_2_D_3_ dramatically suppressed the migration and invasion in both M-I and M-L cells. Furthermore, 1α,25(OH)_2_D_3_ decreased the expressions of Vimentin, N-cadherin, β-catenin and Snail in both M-I and M-L cells with stable knockdown of CYP24A1. These results are consistent with a previous finding that CYP24A1 depletion augments the suppressive ability of 1α,25(OH)_2_D_3_ on EMT (17).

The other important finding of this study emerges from the phenomenon that knockdown of CYP24A1 increased the levels of E-cadherin in both M-I and M-L cells, and reduced Vimentin (M-L), N-cadherin (M-I), β-catenin (M-L) and Snail (M-I), hinting that silencing CYP24A1 could inhibit EMT. In fact, our results are in agreements with the data described in recent studies (39–41, 44), which suggest CYP24A1 as a potential oncogene. Therefore, it is worth noting whether CYP24A1 would play an oncogenic role through regulating EMT.

In summary, 1α,25(OH)_2_D_3_ partially suppresses the proliferation and invasion of malignant ovarian cancer cells *in vitro* and *in vivo* by inhibiting EMT. Moreover, loss of CYP24A1 not only promotes the inhibitory effects of 1α,25(OH)_2_D_3_ on the migration and invasiveness of intermediately transformed and malignant ovarian cancer cells by inhibiting EMT, but also directly suppresses EMT. These findings provide research foundation for making CYP24A1 a potential target to activate the vitamin D pathway in the treatment of ovarian cancer.

## Data Availability Statement

The datasets presented in this study can be found in online repositories. The names of the repository/repositories and accession number(s) can be found in the article/Supplementary Material.

## Ethics Statement

The animal study was reviewed and approved by Institutional Animal Care and Use Committee of Soochow University.

## Author Contributions

PW, JX, WY, and YH finished the experiments and data analysis. PW and BL designed the experiments. JX, PW, and WH wrote the manuscript and the manuscript was reviewed by BL and ZZ. The funding was acquired by BL, PW, and WH. All authors contributed to the article and approved the submitted version.

## Conflict of Interest

The authors declare that the research was conducted in the absence of any commercial or financial relationships that could be construed as a potential conflict of interest.
