# Supplementary figures and images for: Knockdown of CYP24A1 Aggravates 1α,25(OH)2D3-Inhibited Migration and Invasion of Mouse Ovarian Epithelial Cells by Suppressing EMT
Source: Front Oncol. 2020 Jul 29;10:1258. doi: 10.3389/fonc.2020.01258 (PMC7403498; doi:10.3389/fonc.2020.01258)

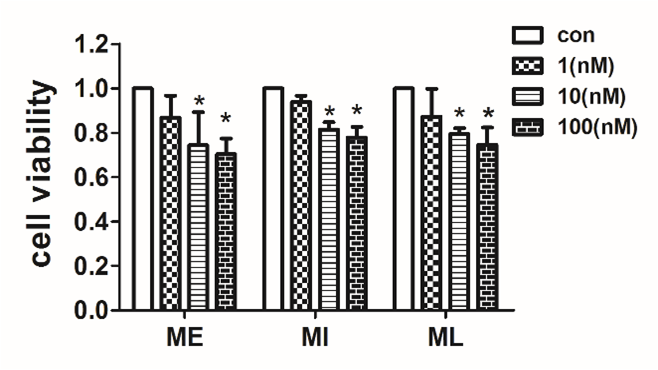

Supplement: Supplementary file 1 [file Image_1.tif]
